# Exploring the perceptions of athletes and DCOs on the remote sampling procedure in anti-doping testing: shifting control to burden

**DOI:** 10.3389/fspor.2026.1825926

**Published:** 2026-06-04

**Authors:** Julian Lanfer, Daniel Westmattelmann, Benedikt Stoffers, Andrea Petróczi

**Affiliations:** 1 Center for Management, University of Münster, Münster, Germany; 2 School for Business Administration, PHWT Vechta, Vechta, Germany; 3 Faculty of Health and Sport Sciences, Széchenyi István University, Győr, Hungary

**Keywords:** anti-doping, athletes, doping control, remote sampling, technology acceptance

## Abstract

**Introduction:**

The approach of remotely collecting anti-doping samples from athletes by utilizing a Remote Sampling System (RSS) presents a technological innovation in anti-doping work, offering a potential solution to the logistical limitations of traditional in-person testing. Even though the implementation decision obliges Anti-Doping Organizations (ADOs), successful implementation hinges on whether athletes as the key stakeholders of anti-doping work perceive an RSS as legitimate. Furthermore, the perceptions of DCOs who are the key users operating an RSS and having to adapt to new sample collection procedures are largely unexplored. This study adopted an exploratory qualitative design to investigate the perceptions of athletes and DCOs.

**Methods:**

We conducted semi-structured interviews with 16 elite athletes and 22 professional DCOs. Data were analyzed using template analysis to identify their key themes and considerations associated with procedural steps of remote sampling applied in anti-doping testing.

**Results:**

Our findings reveal challenges in user-system integration, which we conceptualize as a shift from DCO control to athlete burden. For athletes, the transfer of specific sample-handling responsibilities inherent to the remote sampling procedure is perceived as an unjust burden of personal risk, logistical effort, and procedural anxiety. Key concerns include the management of test kits and the liabilities associated with taking responsibility for unobserved sample shipment. Conversely, DCOs interpret this transfer as a critical loss of control over procedural integrity and professional oversight, focusing on the inability to uphold an unbroken chain of custody.

**Discussion:**

The study concludes that RSS implementation is not merely a technological challenge but one of trust management that must consider user perceptions and concerns on how remote sampling can be designed as a legitimate procedure of anti-doping work. It must reconcile the shift from control to burden by simultaneously mitigating the athletes' burden while empowering DCOs with reliable tools, enabling them in their role as guarantors of anti-doping testing's procedural integrity. These insights provide actionable recommendations for anti-doping organizations to pilot new remote sampling protocols that are not only efficient but also perceived as legitimate, trustworthy, and effective.

## 1 Introduction

Anti-doping testing is a cornerstone of international sports governance, designed to safeguard fairness, protect athlete health, and maintain public trust in competitive sport (1). The World Anti-Doping Agency (WADA) and its affiliated Anti-Doping Organizations (ADOs) invest considerable resources into ensuring that athletes compete under doping-free conditions, with the 2027 WADA code and associated International Standards recently being approved (2). More than 100 pages of Standards defined for Testing (3) and 161 pages of Standards defined for laboratories (4) underline that anti-doping testing is meticulously regulated. Here, anti-doping testing carried out by doping control officers (DCOs) collecting analytical samples from athletes can be considered as their core activity, constituting approximately 50% of national ADOs' annual budgets (5, 6). In this traditional model, DCOs contact athletes at their whereabouts to collect urine or blood samples under strict supervision (7, 8), a process central to ensuring both procedural integrity and the deterrence effect of anti-doping work (9, 10).

The COVID-19 pandemic abruptly exposed the vulnerabilities of this system (11, 12). In 2020, international lockdowns and social distancing regulations led to a reduction of in-person testing by nearly 50% (13), generating unprecedented challenges for the global anti-doping community (14). In response, several ADOs in collaboration with technology providers launched pilot projects to test the feasibility of Remote Sampling Systems (RSS) that allow athletes to provide samples from their homes under virtual supervision (15–17). Early pilot projects demonstrated multiple potential benefits of RSS. On the one hand, financial and ecological savings were explored, with fewer DCOs traveling leading to lower travel costs and significantly reduced carbon emissions (18, 19). For example, the German National Anti-Doping Agency (NADA), in cooperation with sports technology provider Sportradar, reported that 288 remote tests conducted during a five-month pilot reduced travel by 20,000 kilometers and cut emissions from 3.25 tons to 0.02 tons of CO₂ equivalents (20). These developments suggest that RSS could represent more than a temporary response to pandemic restrictions, but instead a long-term tool to enhance sustainability and effectiveness in global anti-doping testing (14, 21).

**Figure 1:**
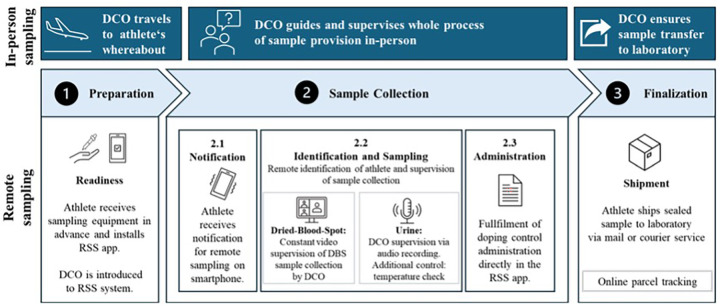
Remote sampling process compared to traditional in-person sample collection. Note. In accordance with Stoffers et al. (19) and Trinks et al. (20).

Subsequently, a dedicated app-based “Remote Testing System” solution (20) was developed that integrated dedicated, app-based user interfaces for athletes and desktop applications for DCOs, enabling real-time video calls to supervise sample collection. The remote sampling process using the dedicated RSS system is visualized and compared to the traditional in-person sample collection in Figure 1.

The remote sampling procedure can be divided into three sequential phases. The preparation phase addresses technological and procedural requirements, including the advance delivery of sample collection kits and the athlete's introduction to the remote testing app. Also, DCOs are introduced to and trained in the remote sampling procedure and application. Once remote sampling readiness is achieved in step 1, the sample collection can be triggered by a notification that athletes receive on their smartphones, followed by identity checks under video supervision. The actual sample collection is then supervised via the remote sampling app, with Dried Blood Spot (DBS) samples being video-supervised utilizing self-collection devices such as TASSO (22), while urine sample collection may involve audio monitoring and temperature controls to protect privacy (14). After that, doping control administration associated with sample collection is conducted within the app. Lastly, the finalization phase completes the process through initiating tracked shipment of the collected sample to an anti-doping laboratory, ensuring adherence to chain-of-custody regulations (23, 24). Two organizational features of these pilots are worth noting. First, shipment costs of remote samples are borne by the ADO, so that athletes do not incur direct financial costs. Second, these pilot projects operated outside formal testing frameworks: irregularities such as samples not arriving at the laboratory could only inform decisions on subsequent target testing, while rules governing athlete liability under a fully implemented RSS remain to be specified in dedicated remote sampling guidelines.

### 1.1 RSS technology adoption in the sensitive context of anti-doping testing

While the technical feasibility of remote sampling has been demonstrated, the remote procedure introduces profound challenges due to the sensitive context of anti-doping testing. Unlike in non-mandatory settings like telemedicine, the anti-doping context involves high stakes: athletes tested positive in doping controls face bans of up to four years or even criminal prosecution, having the potential to end their sporting career (25–27). This raises heightened concerns about sample integrity, chain of custody, and the procedural trustworthiness required to meet the rigorous evidentiary standards of sports arbitration (14, 28). Existing research on RSS remains fragmented. Organizational-level studies indicate that ADO leadership endorses RSS for its efficiency gains and resource savings, but expresses regulatory concerns particularly about compliance with WADA's strict integrity standards (8, 19). Analytical research highlights the promise of DBS sampling for remote applications but does not explicitly address user acceptance in a remote setting (29, 30). While these strategic and technical perspectives are valuable, they overlook the “lived realities” of the system's primary users, namely athletes who must comply with testing regimes to pursue a career in professional sports, and DCOs, whose work is fundamentally altered (31). A critical gap thus exists in understanding the perceptions of users as key frontline stakeholders of remote sampling adoption.

### 1.2 Remote sampling legitimacy

The implementation of RSS occurs in a mandatory use environment, a crucial context that shapes user perceptions. Athletes cannot refuse testing without risking suspension, and DCOs are obliged to follow prescribed procedures as part of their professional responsibility (8, 32). Research on technology adoption shows that in such mandatory settings, user acceptance hinges less on voluntary choice and more on perceptions of legitimacy, fairness, and trust (33, 34). Negative perceptions can fuel resistance or non-compliance, which in the context of anti-doping can undermine the very integrity an RSS seeks to protect (35).

While organizational perspectives on RSS implementation have been investigated (19), systematic qualitative insight from athletes and DCOs as the actual users of an RSS is lacking. As the central stakeholder group, athletes' perceptions of legitimacy, fairness and trust directly influence the deterrent effect of testing regimes (7). In the context of anti-doping and remote sampling implementation, legitimacy is typically understood as perceived legitimacy. This emphasizes that both athletes and DCOs must believe that remote sampling is an appropriate, proper, and just procedure in anti-doping work (36, 37). If athletes perceive RSS as unreliable or unfair, it could erode their perception of legitimacy of the entire anti-doping system (38), while significant privacy concerns remain to be explored (39). DCOs, by contrast, are the operational backbone responsible for procedural integrity (8). RSS fundamentally alters their work routines and professional identity by shifting responsibilities and increasing reliance on digital tools (31, 40). Securing their endorsement is therefore crucial, as a lack of trust and perceived legitimacy of remote sampling could lead to procedural lapses that compromise testing integrity (35).

Technology users provide critical information on e.g., perceived benefits, risks, and trust factors (41) and highlight practical challenges and uncertainties that may not be visible at strategic levels of implementing testing regimes. This study addresses this gap by providing in-depth qualitative insights into their views, which are critical for a comprehensive evaluation of RSS feasibility (42). The aim is to inform ADOs whether, and under which conditions, RSS can become a legitimate component of the anti-doping system. To do so, this study pursues an exploratory qualitative approach (43) to investigate athletes' and DCOs' perspectives on RSS implementation. Specifically, this study seeks to answer the following research questions:

**RQ1:** What specific challenges and opportunities do athletes and DCOs identify across the three key phases of the remote sampling process (preparation, sample collection, and finalization)?

**RQ2:** Where do the perspectives of athletes and DCOs converge or diverge, and what are the implications of these differences for the successful implementation of RSS?

Through template analysis (44), the gathered qualitative interview data is structured along the key steps of the remote sampling procedure [preparation, sample collection, and sampling finalization; (19)], enabling a comprehensive and process-oriented understanding of user perceptions of remote sampling (42). Moreover, by explicitly comparing the perspectives of athletes and DCOs, the study highlights converging as well as diverging views on implementing an RSS in anti-doping testing regimes.

## 2 Materials and methods

### 2.1 Research context

This study is situated within a broader research initiative titled “Implementing a remote sampling system in anti-doping work,” supported by the Partnership for Clean Competition (PCC). The overarching aim of this project is to conduct a multi-stakeholder investigation into the feasibility, benefits, and risks of integrating RSS into global anti-doping programs. By focusing on the perspectives of primary users (elite athletes and DCOs), the project seeks to understand the conditions under which digital innovations can supplement traditional testing while maintaining procedural integrity and stakeholder trust.

The project follows a sequential study design. The current qualitative study represents the first phase, utilizing semi-structured interviews to capture the “lived realities” and granular, process-oriented concerns of athletes and DCOs. These foundational insights provided the empirical basis for a companion quantitative paper, which statistically validates the themes identified here to compare perceptions of legitimacy and attitude across larger user cohorts. By first exploring the subjective experiences of stakeholders in this paper, we ensure that the subsequent quantitative modeling remains contextually grounded in the actual procedural challenges of remote sampling procedures.

### 2.2 Research design

This study adopted a qualitative research design. Through in-depth interviews, the perceptions, experiences, concerns, as well as opportunities and challenges perceived by athletes and DCOs as RSS users were explored (43). Given the lack of prior empirical work on user perceptions of remote sampling systems, a generic qualitative approach was deemed most appropriate (45). Qualitative inquiry is particularly suitable for this research, as it allows for a systematic exploration of the subjective meanings and situated practices associated with the use of novel procedures or technologies, like RSS implementation in anti-doping testing (46, 47). By focusing on the perspectives of athletes and DCOs, the study aimed to capture how RSS users make sense of this novel procedure and how they perceive its implementation across the different remote sampling process stages (19).

The study was guided by a pragmatist paradigm, which prioritizes the research problem and the practical consequences of inquiry, focusing on generating knowledge that is useful for action in real-world contexts (48, 49). From this perspective, ontology is treated as pluralistic and context-dependent, approaching “reality” through what proves workable and meaningful in a given situation rather than through commitment to a single, fixed account of the world (43). Epistemologically, pragmatism supports producing warranted knowledge by using methods best suited to the research question and by evaluating insights in terms of their explanatory and practical value for informing decisions (48). Accordingly, the qualitative interview approach was used to derive practice-relevant insights into how athletes and DCOs perceive and navigate RSS procedures, with the aim of informing implementation in anti-doping practice.

### 2.3 Sample and interview procedure

For this study, purposive and snowball sampling were employed to recruit participants who could provide detailed insights to address the research questions (50, 51). Based on the inclusion criteria, the sample comprised participants who were: (a) over 18 years old, (b) fluent in German or English, and (c) either current or recently retired elite athletes from registered testing pools (RTP) or national testing pools (NTP), or professional DCOs affiliated with National Anti-Doping Organizations (NADOs) or international testing agencies.

The final sample comprised 16 elite athletes and 22 DCOs. Athletes (5 female, 11 male) were recruited from various high-doping-risk sports categories (24) and included Olympic and Paralympic medalists as well as world and European champions across various summer and winter disciplines. DCOs from seven countries were recruited, including interviewees from Germany (15), Italy (2), Belgium (1), Brazil (1), Greece (1), Ireland (1), and Spain (1). Both DCOs with prior remote sampling pilot experience (*n* = 6) and those with exclusively traditional testing experience (*n* = 16) were recruited. Athletes were recruited through direct contact via sporting federations, personal networks, and snowball sampling techniques (50). DCOs were recruited through one NADO and two international testing agencies. All participants received comprehensive information about the study objectives, data protection measures, and provided informed consent prior to participation.

Semi-structured interviews were chosen as the data collection method due to their ability to combine systematic coverage of predetermined topics with sufficient flexibility to follow participants' accounts and probe emerging issues (46, 52). Interview guides were developed through an iterative, collaborative process among the research team, incorporating feedback from anti-doping experts and pilot testing with representative participants (42). A “mirror” format was employed, enabling parallel questions for both participant groups while allowing for group-specific probes (53). The guides were structured around the three main stages of the RSS procedure (preparation, sample collection, and finalization), complemented by sections addressing overarching constructs such as trust, transparency, procedural justice, and technology acceptance (54, 55).

Prior to each interview, participants received explanatory materials including a process diagram and short video demonstrating RSS procedures to ensure a shared understanding of the technology (42). All interviews were conducted via secure online video conferencing platforms between February and October 2023, lasting between 45 and 90 min. The online format was chosen to accommodate participants' geographical distribution and schedules while maintaining privacy and confidentiality (56). Interviews were conducted by experienced anti-doping researchers in German and English, audio-recorded with permission, and transcribed verbatim. All transcripts were anonymized. Data collection continued until the absence of new themes or insights from additional interviews was perceived by the researchers (57, 58).

### 2.4 Data analysis

Data analysis employed template analysis, a style of thematic analysis that uses a coding template which is refined during engagement with the dataset (44, 59). This approach was chosen because it provides an analytic structure while remaining flexible, allowing the analysis to be oriented toward the practical research problem and the study's pragmatist positioning (43, 44). The output of the template analysis captures recurrent and relevant themes of participants' accounts in relation to the research question, incorporating *a priori* structuring where appropriate (44, 59). In this study, the three RSS process stages (preparation, sample collection, finalization) were used as structuring and organizing elements to ensure a process-oriented and implementation-relevant analysis. Within this structure, themes were developed inductively from the interview material. Rather than deriving *a priori* themes from literature, the RSS process steps (19, 20) served as *a priori* structural framework. This pragmatist choice proved analytically useful for organizing inductively derived codes and themes in direct alignment with the study's research questions.

Following guidance on template analysis, the analytic procedure comprised: (1) familiarization with transcripts through repeated reading; (2) initial coding of material relevant to the research questions within the a-priori template structure of RSS process stages; (3) iterative modification of the codes within the template structure as additional transcripts were coded; and (4) definition of a final theme map that could accommodate all relevant data and support write-up of qualitative results (44, 59). Throughout coding, only explicit, that is, directly spoken, content was coded, as implicit meaning was considered beyond the scope of the pragmatist and applied orientation of the study. Coding proceeded sequentially, beginning with athlete transcripts and then moving to DCO transcripts, which enabled explicit examination of convergences and divergences between the two user groups within the shared RSS process structure (44, 59). Initial coding and theme development were conducted by the first author, with continuous input from the co-authors across both the athlete and DCO coding phases. Tables 1, 2 present the applied coding template for both the athlete and DCO interviews.

**Table 1:** Developed coding template, athlete perspective.

Perspective	Remote Sampling Step	Developed Theme	Interview Code
Athlete Perspective	Remote Sampling Preparation	A1. RSS readiness: Equipment and technology challenges	Kit availability
			Kit delivery
			Kit security
			Device requirements
			Technical instructions
		A2. Test announcements and whereabouts transitions	Pre-notification test scheduling
			Travel & location challenges
	Remote Sample Collection	A3. RSS intrusiveness and privacy	Pain & invasiveness
			Privacy concerns
			Post-notification reaction
		A4. Remote supervision: technology and tampering security	Supervision equipment reliability
			Video supervision considerations
			Tampering risk
			Analytical sample checks
		A5. Self-sampling and associated support needs	General sampling support needs
			Para-athlete support
			Self-sampling usability
			Self-sampling responsibility
	Remote Sampling Finalization	A6. Digital convenience	Digital convenience
		A7. Shipment and logistics: responsibility and liability	Drop-off solutions
			Sample loss and tampering risk
			Shipment insecurities/intransparency
			Tracking & proof need

**Table 2:** Developed coding template, DCO perspective.

Perspective	Remote Sampling Step	Developed Theme	Interview Code
DCO Perspective	Remote Sampling Preparation	D1. Kit delivery: logistics and advance-notice risks	Advance notice concerns
			Delivery risk
			Shipment trackability requirements
			Kit dispatch planning
			Kit quantity planning
		D2. Kit integrity issues	Athlete kit storage and handling responsibility
			Kit integrity prerequisites
			Kit expiry tracking
		D3. DCO work environment: readiness training and new work reality	RSS tech training
			Technical equipment requirements
			Cost savings
			DCO workload savings
	Remote Sample Collection	D4. Balancing athlete identification, cooperation, and privacy	Athlete verification
			Athlete cooperation
			Privacy concerns
		D5. DCO supervision quality and technological demands	Camera positioning
			Continuous visibility of provision
			Athlete welfare
			Sample sealing and tampering risk
			RSS system reliability
	Remote Sampling Finalization	D6. Roles & responsibilities redistribution	Athlete custody & responsibility
			DCO responsibility scope
			Courier handling & shipping
		D7. IT administration and chain-of-custody documentation	Chain-of-custody documentation
			IT administration
			Chain-of-custody regulations clarification

### 2.5 Methodological rigor and quality criteria

Consistent with the study's pragmatist positioning, quality assurance focused on producing a well-documented analysis that is both analytically credible and useful for informing practice (43). Credibility was supported through sustained engagement with the dataset, iterative team discussions during template development, and systematic comparison across participant groups (athletes and DCOs), allowing competing interpretations of codes and themes to be challenged and refined (44). Reflexive notes were used to make analytic assumptions visible and to support critical examination of how the research team's prior knowledge of anti-doping contexts shaped the development and interpretation of themes (44, 59). In particular, themes that could not be clearly attributed to one of the three RSS process steps were either modified or removed following team discussion, ensuring that the final template remained structurally coherent. Transferability was facilitated by grounding interpretations in participants' accounts through illustrative quotations, enabling readers to judge the applicability of findings to other (anti-doping-) contexts (44).

## 3 Results

**Figure 2:**
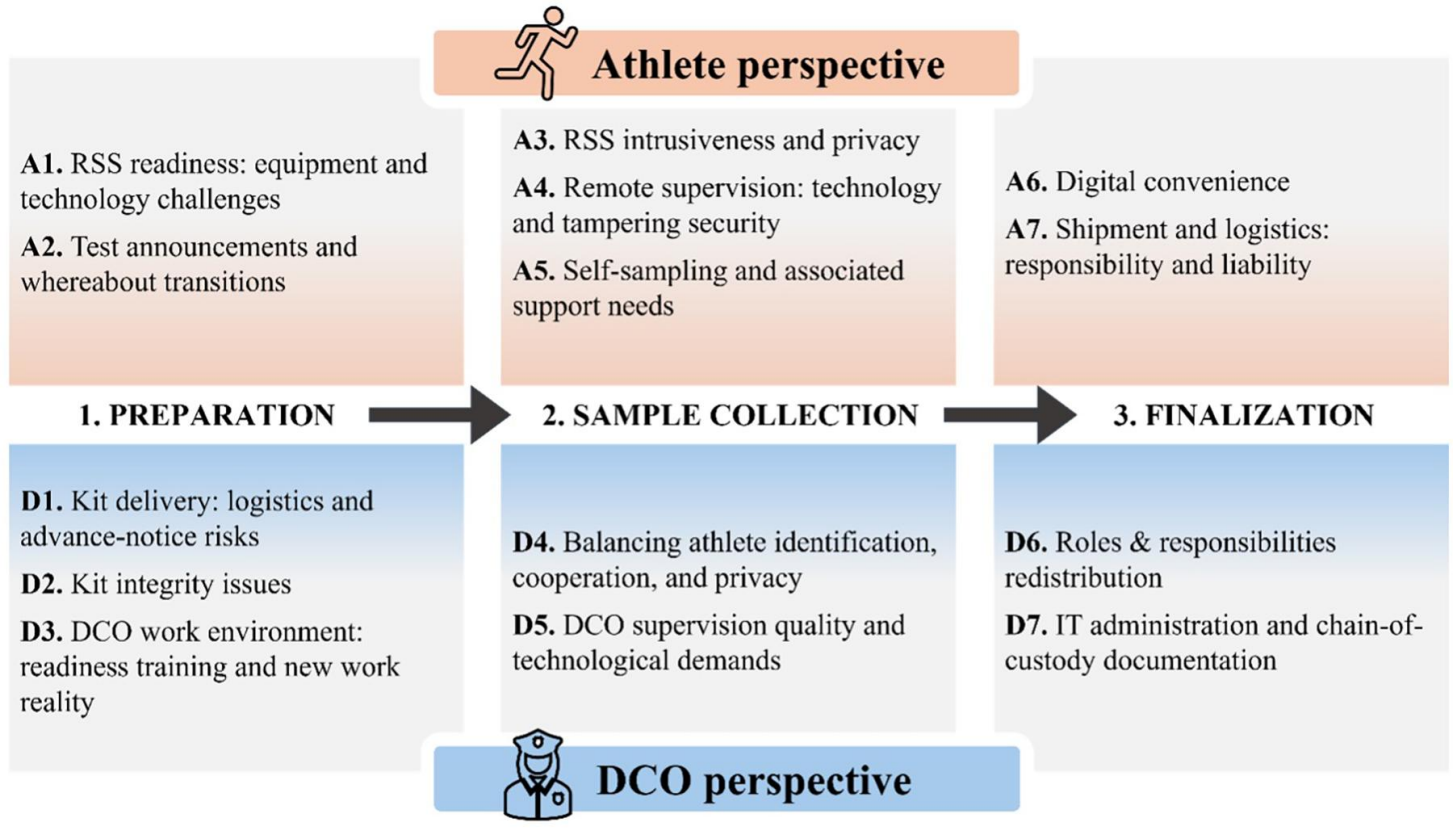
Identified athlete and DCO themes within the remote sampling process steps.

The interview material was systematically coded and organized into overarching themes that capture key aspects of athletes and DCOs perspectives on remote sampling. Within the analysis template, themes were aligned with the three sequential process steps of the remote sampling procedure: Preparation (Step 1), Sample Provision (Step 2), and Finalization (Step 3) (19). Within each step, distinct themes reflect specific challenges and perceptions of athletes and DCOs. To account for the dual perspectives of both user groups, themes were differentiated between both user perspectives and are visualized and juxtaposed in Figure 2. This allows for an overview and nuanced analysis that highlights both converging and diverging viewpoints regarding e.g., technological feasibility, procedural integrity, and user experience across the different stages of the remote sampling process. The following results section presents the identified themes divided into the three process steps of remote sampling, supported by selected, illustrative interview quotes from athletes and DCOs.

### 3.1 Remote sampling preparation

In the preparation phase, athletes and DCOs both frame remote sampling as a logistics-heavy shift in responsibilities, but they evaluate the consequences through different lenses: Athletes focus on day-to-day feasibility to achieve RSS readiness as well as associated personal risk, while DCOs focus on procedural integrity, operational control, as well as anticipated shifts in their working routines.

***A1. RSS readiness: equipment and technology challenges***

A central concern for athletes in the preparation phase is the permanent availability of testing kits. The obligation to always have a testing kit at hand creates a significant burden. Athletes describe the difficulty of carrying the kit at all times and note associated challenges. They are concerned about kit mishandling due to, for example, having to change it between travel luggage or training bags. For athletes, this leads to the fundamental question of how immediately the material must be available.

[“(…) do you really have to carry it around with you all day, everywhere you go? Because that’s obviously annoying if I have to remember to take it with me to every single training session, to the physio and doctor’s appointments, to work and everywhere else. (…) I actually organize myself so that I use different bags for many things. But then I always have to remember that it has to be transferred from one bag to the next when I go to training or work or depending on what I need at the moment.” (AD18)]

Besides kit availability, athletes are concerned about kit delivery and its security. Delivery by post or courier is described as practicable, but also entails risks such as missed deliveries, for example, when neighbors accept packages due to not being at home. Here, athletes emphasize that training camps or extensive competition schedules in remote places complicate reliable, personalized delivery of remote sampling kits. Adequate storage conditions and shelf life of kits are further sources of uncertainty. Concerns regarding technological requirements focus on the constant need for a stable internet connection and a constantly charged and available smartphone.

[“[I see the problem] that you have to be reachable via your smartphone. (…) I really don't know what [the DCO] would do if you weren't reachable at all. Whether there would be any consequences right away or whether he would just come back the next day. But then you would have to see what kind of tolerance you have in the time between receiving the notification and actually looking at it, what kind of tolerance you have set.” (AD10)]

***A2. Test announcements and whereabouts transitions***

In light of testing kit delivery, athletes are uncertain whether the act of receiving a kit can be interpreted as a de facto announcement of an impending test. They feel like remote sampling preparation might undermine the principle of no-advance-notice testing, which is connected to the obligation of whereabouts reporting by athletes.

[“But I do wonder a little bit: if an athlete receives the items in advance, then it’s kind of like an announcement. And now, athletes who have something to hide could react accordingly. So isn't that why the concept is that the (ADO) suddenly shows up at the door unannounced?” (AD24)]

In this regard, athletes anticipate that remote sampling adds an additional burden to current whereabouts reporting routines, as they expect to have to report not only whereabouts for traditional testing, but also time slots they are able to be available for remote testing. Here, athletes voice that adequate reaction timeframes are necessary to make remote sampling possible:

[“But I would just say something, something between 4 and 8 h maybe. That you somehow have a certain time frame to prepare yourself, to possibly, yes, get to a place where it’s feasible, where it’s possible. You can't make a video call everywhere right now. So that you have a little bit of a chance to get yourself into a suitable position. But then still, don't make it too long. I think the same day is definitely good, and then, yes, not much longer than, yes, 4 to 8 h maximum. So that it’s reasonably close to the original check-up.” (AD13)]

***D1. Kit delivery: logistics and advance-notice risks***

From the DCO viewpoint, the logistical processes necessary for reliable remote sampling are prone to failure. Resonating with the themes voiced by athletes regarding kit delivery, DCOs anticipate lost packages, delivery problems due to incorrect addresses or athlete mobility, or significant delays caused by customs. For reliable delivery, DCOs consider personal handover with a signature and seamless shipment tracking necessary.

[“We have the problem that many athletes do not link their contact address, i.e., their postal address, to their home address because they are simply on the road a lot. This means that the material may not be where the athlete is. (…) It could possibly be a problem, but the athlete would then have to arrange how he can get the documents, the material, in a timely manner.” (DCO11)]

[“[The athletes] have to accept the delivery. Maybe they're not there. It doesn't get delivered. The package gets lost, lots of issues with just getting the sample equipment to the athlete. (…) And they say it’s an accident. It’s really flimsy.” (DCO08)]

Moreover, DCOs anticipate that the delivery of kits to athletes poses problems. Same as athletes, DCOs voice that it potentially undermines the principle of no-advance-notice testing. If testing is tied closely to kit delivery, it is causing the surprise element to disappear.

[“Well, first of all, if there’s a high-level athlete, they know they'll be tested sometime. But when the equipment arrives at the house, they know it’s gonna be soon, probably (…) like, I know they’re now coming within a month, two months (…) when they do get the equipment, it’s just a little bit more ‘Ohh, they're definitely going to come. They're definitely going to take a sample!” (DCO08)]

***D2. Kit integrity issues***

A central theme for DCOs is the integrity of the test kit once it is in the athlete's custody. The fact that the equipment is unattended not only with the athlete, but also with third parties like courier services leads to fundamental questions about whether the kit is suitable for anti-doping testing. Even though some DCOs are less concerned due to the kit being numbered and sealed tamper-evident, the concern remains that athletes with malicious intent could find ways to manipulate them. The responsibility for the storage and current handling of the kit lies entirely with the athlete, which is viewed critically by DCOs. A further voiced practical problem is the potential expiration date of the kits. DCOs highlight the administrative effort required to monitor kit expiration and send replacements in time.

[“(…)You should always assume that the athlete is interested in submitting a proper doping test and cooperating. And in that respect, I wouldn't have any problem with the athlete receiving the kit on their own. (…) But the fact is that we don't let our materials out of our sight at all. (…) honestly, if I were to tamper with it using a small syringe or needle or something, I wouldn't be able to see from the outside on the screen whether anything had been tampered with or not. Whether there was a small hole or anything else. So, I don't know if we should underestimate how important it is that we keep an eye on our material.” (DCO22)]

***D3. DCO work environment: readiness training and new work reality***

DCOs unanimously emphasize the necessity of comprehensive training before using the RSS. This includes both familiarization with the technological platform and practicing the actual process, which is by some DCOs considered as easier than the in-person procedure. The need for explanatory videos was seen as particularly important for less tech-savvy colleagues.

[“(…) the preparation, the training is very, very important. I just finished a project (…) where we created an e-learning program for the DCOs who will work at the Olympic Games (…). I believe it is really, really important to train DCOs about these new platforms. But again, I believe that with good training, all the DCOs would be ready to switch. Because they are switching to something easier, actually.” (DCO04)]

[“But basically, I believe that it wouldn't be a bad idea, especially for the older colleagues, if there were some kind of explanatory video beforehand, or even several. Or just a complete training course on it. I think that is a must, because they still have problems with our control method from time to time.” (DCO19)]

At the same time, DCOs recognize clear advantages in the new workflow associated with remote sampling. The most significant change is the elimination of travel time and costs, leading to a considerable reduction in workload.

[“It would be advantageous for me in terms of processing, as I would not need to travel anywhere. I would not need to consider how much advance notice I would need to leave in order to reach an athlete, particularly if they have a so-called one-hour slot, within the time I need to check them. I would not need to load equipment, pack it, or ensure in advance that everything is in order (…). That would certainly be a definite advantage. I could handle everything just as we are communicating here now, with the relevant athlete.” (DCO14)]

However, the focus shifts to new technological requirements that must be prepared, such as being able to assist in case of technical issues such as ensuring a stable camera setup, or a clear audio connection.

[“I expect that in the remote setting for example, you need to give more instructions to the athlete, put the camera there, not the camera there, there is not good, bring something to put the camera on, bring a table (…). You need to give more instructions, it would be more time-consuming (…). You still have the responsibility to say no, this is not good, no, now I cannot see, I cannot see because the camera is not in a good position, because you're not standing in the right position (…). So you still have to guide the athlete through the procedure.” (DCO01)]

### 3.2 Remote sample collection

From the athletes' perspective, the sample collection phase is primarily defined by whether remote supervision can keep the procedure practically manageable in real-world settings. From the DCOs' perspective, the sample collection phase is a fragile compromise where feasibility, supervision quality, and manipulation risk are in constant tension.

***A3. RSS intrusiveness and privacy***

Viewed from a privacy perspective, athletes perceive remote proctoring techniques as advantageous compared to traditional in-person proctoring of sample collection due to not having a DCO present in their own home as a personal, private space. Still, diverse possibilities of remote proctoring (e.g., audio recordings, live video, or video recordings) introduce uncertainty about which technology is “good enough” to serve its purpose without being perceived as too intrusive.

[“So, what I found most unpleasant about the home check was actually always having strangers in my apartment (…) they have more or less invaded my space and usually don't show as much consideration as guests would. So yes, then the dining table has to serve as a place to transfer the urine sample and so on, and that's always a bit, well, difficult, because maybe you didn't have time to tidy up the night before, you ate late, everything isn't tidy and so on, and you feel a bit uncomfortable. And, and this, this aspect, you wouldn't have that at all anymore. So you could sit down wherever you want.” (AD23)]

Athletes' evaluations of proctoring intrusiveness depend strongly on the sample type that is collected during remote supervision. In a remote setting, urine sample provision is experienced as even more problematic than in in-person settings, surpassing a privacy threshold due to the potential of video cameras capturing and recording intimate bodily functions. It should be noted that video supervision of urine collection is not part of currently piloted RSS procedures, which rely on audio monitoring (cf. Figure 1). Athletes nonetheless raised this scenario unprompted to mark the boundary of acceptable remote oversight. In contrast, DBS sampling is perceived highly acceptable and described as a low-burden method compatible with remote video proctoring.

[“Honestly, (…) I would have a bit of a problem with data protection, because you'd have to film everything. I don't know if I'd want that, you know what I mean. If you film me on the toilet, I personally wouldn't feel comfortable with that.” (AD11)]

***A4. Remote supervision: technology and tampering security***

Even though athletes widely acknowledge and accept video supervision as the core integrity mechanism of remote sample collection, they perceive the integrity promise of video supervision as fragile and dependent on the DCOs' rigor in supervision, as one athlete that participated in a remote sampling pilot project points out:

[“(…) as a DCO, you would definitely have to make sure that you can see everything in the picture at all times and not have your hands so low that the camera can no longer capture them. I think that’s feasible, (…) you just have to pay attention to it. I remember being surprised that you couldn't always see everything I was doing in the video. (…) Maybe there are some clever ideas on how to ensure that the DCOs really do make sure that everything is visible in the image.” (AD13)]

The possibility of manipulation by individuals with such intent remains a central concern to many athletes: They fear that sample vessels could be prepared in advance or seals could be faked with sophistication, especially if such individuals have the sampling material constantly at hand. Analytical checks in the laboratory are seen as a counterbalance to these procedural uncertainties, even though they are perceived as lacking transparency.

[“Of course, it’s a little harder to imagine at first glance that the whole thing can't be tricked. Because if you have the materials permanently available to yourself, isn't there some way to forge the seals and ultimately submit things incorrectly anyway? On the other hand, I mean, it’s true that you can identify afterwards whether the blood and urine are your own. (…) But can you really verify that it really came from that time? Or could it have been from two weeks before or a year before?” (AD18)]

***A5. Self-sampling and associated support needs***

The self-collection of the sample by the athlete is described as feasible but requires adaptation. The DBS test procedure was perceived by athletes that were part of remote sampling pilots as “*pretty foolproof*” (AD15). Nevertheless, it is acknowledged that a certain time to adapt to the new sampling procedure is necessary. A high need for support is seen, especially for athletes without experience, for example through explanatory videos or personal guidance. A specific concern relates to para-athletes. Athletes raise the question of how impaired athletes are treated and what happens if no support person is available, highlighting the necessity of developing inclusive and adaptable protocols.

[“And what I'm wondering now is how this works for some para-athletes who need help in some way. How do you do that? (…) For us visually impaired and blind people, there always has to be someone with us. (…) I don't know if I could manage everything on my own if there was only one camera in front of me. And I also don't know how it’s done for athletes who may have some kind of disability in their hands or spasticity or something like that.” (AD24)]

***D4. Balancing athlete identification, cooperation, and privacy***

Identifying the athlete via video is described as error-prone. DCOs pointed out issues with outdated ID photos and difficulties in reliably identifying individuals from various cultural backgrounds.

[“If they have a brother or sister with whom they look alike. And the other thing is that, I'm thinking of this now, they should have an updated photo on the document that they will show us. Because if they have a photo with short and blonde hair and all with beard and moustache, or a photo since seven years ago(…) no way that you can tell the difference. Very often when they are in front of you (…) you have to look again and again to identify the person to make sure that they are the right person.” (DCO01)]

DCOs express concerns about data protection laws and same as athletes, DCOs perceive video supervision of a urine sample as an extreme invasion of privacy. Remote identification and sample provision is seen as fundamentally reliant on the athlete's cooperation. This dependence on trust is a source of professional unease for DCOs. Furthermore, DCOs feel unable to ensure the athlete's well-being remotely. In case of medical issues that can occur during sample collection, such as an athlete fainting, one officer worried:

[“On the screen, I just see him collapse. And then what? I can't call for help” (DCO11)]

***D5. DCO supervision quality and technological demands***

According to DCOs, the quality of remote supervision is highly dependent on reliable proctoring technology. First and foremost, camera positioning is crucial to avoid blind spots, as DCOs emphasize that they can likely only see a small frame, but not the athletes' surroundings. The need for continuous, uninterrupted visibility is paramount in DCOs' considerations, pointing out that DCOs must be able to judge when supervision quality is sufficient.

[“(…) this video image, you only see the excerpt, just like here [in the interview]. You don't know what’s happening around (…) even if they always try to show it, you never have the complete picture, so what do you want? Do you want to see everything (…) or do you just want to see a small section, but you don't know what’s happening in front of you or around you. So that’s one thing I think needs to be worked on with this video image section. Especially when the athlete has to actively type something on their cell phone or something. It’s just, yes, it’s just the question of close-up or long-distance image.” (DCO15)]

System reliability is a major concern for DCOs because it is critical to procedural integrity, with issues like connection dropouts or frozen video feeds threatening the integrity of the test. Any interruption could force a test to be aborted, as one DCO noted:

[“If the technology fails the moment he presses the [DBS] device onto his arm, then the test is invalid.” (DCO11)]

### 3.3 Remote sampling finalization

In the finalization phase, the advantages of digital processing come to the forefront but are, especially from the athlete's perspective, contrasted with perceived risks and uncertainties regarding sample shipment.

***A6. Digital convenience***

Within the RSS system, athletes perceive the sampling finalization as more efficient and flexible due to the use of digital tools: They especially perceive time savings through the digital system, as well as increased convenience due to the elimination of manual signatures and having documents read aloud and signed in-person.

[“One huge advantage is simply that anyone can do it on their smartphone or computer. They don't have to sit at a table next to the DCO, but can simply fill it out comfortably in bed or wherever they happen to be. Somewhere in a café or at the swimming pool or anywhere else. So again, it’s much, much more flexible. And it also saves time, yes. That’s another huge advantage.” (AD24)]

***A7. Shipment and logistics: responsibility and liability***

In the finalization phase, a major uncertainty of athletes concerns the sample shipment. Athletes feel that increased responsibility for sample integrity lies with them. The fear for sample loss and unclear liability are central concerns. Especially, uncertainty arises about what happens after handing the sample over to a third-party postal service or courier, which is considered a “logistical black box”. These concerns are especially present when athletes feel responsible for having to ship samples from remote places with unfamiliar logistics procedures, for example during training camps.

[“There is simply an enormous amount of responsibility on the athlete, which he cannot always fulfill. For example, training camps in Tenerife on Mount Teide, at the top of Mount Teide. (…) Up there they have to submit a sample and then they have the sample up there at 2,300 meters. So, yeah, shit, yeah. Now they have to go to a post office. Switzerland is still okay. But, yeah, Teide or they're somewhere up in Colombia. Yeah, find a post office there (…) it’s virtually impossible.” (AD26)]

The uncertainty leads to a strong need for tracking and confirmation to reduce uncertainty. Athletes demand detailed shipment tracking for their packages and clear proof of drop-off. Also, various shipping solutions are discussed. While some see the trip to the post office as an additional effort, others prefer a courier pick-up as more convenient. The use of automated parcel stations is also mentioned as a convenient alternative.

[“(…) so I would also say that a courier has to come by, because otherwise you have the extra work of going to the post office again, and (…) to make it as easy as possible for the athletes (…). I could imagine that some athletes (…) who are not so conscientious or responsible (…) might then slack off a bit or (…) might not send it off (…) because, well, the question is, what happens if you don't send it? Is it a strike, is it a positive test?” (AD15)]

[“Once I have a confirmation from the place where I dropped it off, I'm no longer concerned about the sample.” (AD26)]

From the DCO perspective, the finalization step is seen as a process of responsibility transfer and a test of the system's legal and logistical robustness.

***D6. Roles & responsibilities redistribution***

For DCOs, the most central theme of the finalization phase is the shift of responsibility to the athlete. DCOs voice that once the video connection is terminated, full responsibility to handle and ship the sample correctly lies with the athlete. This shift of responsibility is viewed ambivalently by the DCOs. On the one hand, they perceive it as a relief from work and a gain of convenience.

[“Wonderful. Less work is always good. No, I mean it’s also a lot of effort (…)and there are 1,000 things to keep in mind and that would all be eliminated. Wonderful.” (DCO21)]

On the other hand, they relinquish control over a critical part of the chain of custody. Consequently, DCOs clearly redefine the boundaries of their own responsibility, which they see as ending the moment the sample is sealed under their observation.

[“But about the finalization, for us to validate the test, you have to see when the athlete closes the box, puts the sample inside the box, closes the box with tape, puts the bill on top of the box. And then my work is finished. Now, from this time on it is the responsibility of the athletes about the shipment. And we have to ask the athletes if they want to take this kind of responsibility.” (DCO16)]

***D7. IT administration and chain-of-custody documentation***

Complete and legally defensible documentation of the chain of custody is of utmost importance to DCOs, as any case can potentially end up in court. DCOs stress that this digital documentation must be legally sound. They emphasize that the rules for a remote chain of custody must be clarified with WADA and that clear policies must exist, for example, when a sample is lost. Without a firm set of rules, the legal validity of the entire process remains uncertain.

[“And the other thing that you would need to see is the chain of custody. (…) That is where the sample is at all times from the point of collection until the point it reaches the laboratory. Where it is and in and who is responsible for it, who has access to it. Because if you have positive case in the case goes to court, there would be raised objections who had access to this sample. Who? How many people? 1, 2, 3, 10? What did they do with it? (…) it’s something that has to be also discussed with the WADA who make the rules regarding the chain of custody.” (DCO01)]

## 4 Discussion of results

### 4.1 Athlete perspective on RSS implementation: the calculus of burden

The athletes' perceptions of RSS implementation can be comprehensively understood through the lens of a “**calculus of burden**,” as a consequence of the system's mandatory use context. In this environment, where non-compliance is not an option (32), the central question for athletes is not if they will use the system, but what personal cost in terms of risk, effort, and uncertainties its mandatory usage entails. This perspective aligns with technology adoption research in mandatory settings, stating that perceptions of fairness and legitimacy become paramount when voluntary choice is removed (33). This mandatory frame fundamentally constrains athletes' autonomy. Refusing testing is not a viable option in elite sport, because it effectively forfeits the right to compete (60, 61). Consent in this context is therefore conditional rather than free. This adds to the ethical weight of any new burden the system imposes on athletes, which has already been recognized as substantial (62).

In the preparation phase, the **logistical burden** becomes evident. While athletes do not object to the principle of RSS, the logistical requirement of constant kit availability significantly lowers the perceived ease of use, a key factor in technology acceptance (55, 63). Rather than being a point of outright rejection, this finding offers an insight into RSS design, with athletes' suggestions for receiving multiple kits representing a solution to enhance system usability. Furthermore, the concern that kit delivery acts as a de facto advance notice makes them question the effectiveness of testing conducted through remote procedures, as it likely undermines the deterrent effect of unpredictable testing, which is central to maintaining athlete trust (9, 38).

During the sample collection phase, athletes' perceptions of procedure risks and privacy needs become more acute, creating a **procedural and privacy burden**. While they acknowledge the necessity of video supervision for integrity, they simultaneously question its reliability due to potential technological failures and blind spots. Their strong preference for the less-invasive DBS method over remote urine collection signals a need for balancing mandatory compliance with privacy needs (39). The athletes' preference for DBS sampling aligns with analytical research highlighting the technical feasibility of DBS (29, 30). Taken together, athletes demand a system that is verifiably secure to counteract the perceived procedural and privacy burden.

Finally, the finalization phase demonstrates a trade-off. The appreciation for “digital convenience” confirms the system's potential benefits (63). However, the profound uncertainty of athletes surrounding the unobserved shipping process reveals a **risk burden** perceived by athletes. The “logistical black box” of sample shipment transfers a significant portion of risk onto the athlete, who fears being held accountable for failures beyond their control. These fears are anchored in how disputes typically unfold under strict liability, whereby uncertainties or contested facts are resolved only through formal results management or appeal processes, during which athletes may bear provisional suspensions, reputational damage, and financial losses, even when ultimately exonerated (25, 64). Their strong demand for tracking and confirmation is a call for procedural safeguards that restore a sense of fairness and mitigate the risks imposed upon them by the system. For RSS to be perceived as legitimate by athletes, its design must therefore extend beyond mere functionality to actively mitigate the procedural and logistical risks it imposes on them.

### 4.2 DCO perspective on RSS implementation: the calculus of control

The perspective of DCOs is shaped by their role as guarantors of procedural integrity, viewing RSS through the lens of professional responsibility and risk management (8). It can be understood through the complementary lens of a “**calculus of control**”. As the designated guarantors of procedural integrity (8), their primary focus is whether the technology can uphold the rigorous, non-negotiable standards of anti-doping testing in a mandatory environment. Furthermore, their professional identity is predicated on the ability to oversee and verify every step of the sample collection procedure.

DCOs perceive a **loss of control** in the preparation phase of remote sampling. The moment the kit becomes “unattended” in the athlete's possession marks the first fracture they perceive in the chain of custody, a process they are professionally bound to protect. Furthermore, the DCOs' shared concern about the erosion of the “surprise element” is a professional one, as it removes a key deterrence tool from their arsenal (7). Their demand for training can be interpreted as an effort to regain a sense of control over a changing work environment and maintain high standards within a new technology-assisted workflow, rather than rejecting it (31).

During the sample collection phase, the DCOs perceived **loss of observational control** becomes acute. Their clear rejection of remote urine testing can be interpreted as a defense of their professional standards, as the inability to witness the sample leaving the body is seen as an unacceptable integrity risk. Their unease of having to simply “believe” an athlete in an inherently adversarial context (9) is not compatible with their professional identity. It signals a clear need for technology that augments rather than diminishes the DCOs' ability to verify actions, thereby upholding their professional identity as testing supervisors rather than passive process observers (40). Their conditional acceptance of DBS, based on its superior observability, demonstrates a willingness to embrace innovation where they believe integrity can be maintained.

The finalization phase highlights a fundamental transformation of the DCO role. The ambivalent response to the transfer of shipping responsibility to athletes is seen as a relief from logistical work but also as a loss of **procedural control**. It means they can no longer personally guarantee the integrity of the process from collection to courier. This loss of control could foster a lack of process ownership, potentially leading to the “procedural lapses” that Hsieh et al. (35) warn about. For DCOs, the legitimacy of RSS is therefore directly tied to its ability to provide technological and procedural mechanisms that allow them to maintain a sense of control over the entire process.

### 4.3 Shifting DCO control to athlete burden

By synthesizing these two perspectives, this study outlines a shift from DCO control to athlete burden to understand the challenges of RSS implementation. It reveals that the transfer of responsibility from DCO to athlete is the primary source of conflict, as it is simultaneously perceived as an additional personal burden by athletes and a loss of professional control by DCOs.

Convergence emerges around the advance-notice effect of kit delivery and the logistical insecurities of sampling kit shipping as critical threats to the system's credibility. This shared skepticism about the system's logistical robustness provides a strong message to ADOs and regulators that logistical issues must be resolved for the system to be deemed credible by its users. Furthermore, the shared preference for DBS as a more feasible remote sampling method creates a consensual pathway that aligns user needs with existing analytical research (29, 30).

Divergence lies in the interpretation of responsibility transfer. For athletes, the transfer of tasks is perceived as an unfair burden of personal risk in a mandatory system. For DCOs, it is a loss of professional control and a threat to the integrity of testing regimes. Further divergence lies in trust perceptions. Athletes' trust in RSS technology is focused on procedural fairness and protection from e.g., false positives (“Will the system be fair to me?”), aligning with literature on system legitimacy (38). In contrast, DCOs' trust is focused on system security and invulnerability to manipulation (“Can the system be cheated?”), reflecting their role as institutional guardians (35).

The shift from DCO control to athlete burden is a central challenge of RSS implementation. A system designed solely for efficiency and convenience will likely fail to gain the trust of DCOs, who are its operational gatekeepers. Conversely, a system designed solely for maximum security without addressing the athletes' concerns about fairness, privacy, and logistical burdens will be perceived as illegitimate by those compelled to use it. As highlighted by research in mandatory use contexts, forcing a system upon users without addressing conflicts among user groups can lead to resistance and non-compliance (33), which presents a threat to the integrity of testing regimes. The successful implementation of RSS is not a matter of choosing one perspective over the other, but of designing a system that addresses both. By framing these as system design requirements, ADOs and regulators can focus on how to build an RSS perceived as effective, legitimate, and trustworthy by its users.

## 5 Contributions and implications

### 5.1 Research contributions

This study makes several contributions to the literature on anti-doping, as well as technology adoption. First, it enriches the field of anti-doping research by providing the first systematic qualitative analysis of user perspectives on RSS (47), complementing previous organizational-level studies (19). In doing so, our research directly responds to a high-priority call from the international anti-doping community. The comprehensive Delphi study by Boardley et al. (65) identified “athletes' experiences of anti-doping procedures” and their “place in the anti-doping system” as a key topic for future research. By systematically comparing the two key user groups, we identify a fundamental tension established as a control-to-burden shift, where athletes perceive a transfer of personal risk and burden, while DCOs perceive a loss of professional control and integrity. This conceptualization moves beyond a simple list of pros and cons, offering a structural lens to unpack the complex user experiences that Boardley et al. (65) highlighted as critical to investigate. Our study provides granular, qualitative data necessary to understand precisely these experiences in the context of a technological shift in anti-doping testing, thereby addressing a community-validated research priority.

Second, this research contributes by applying and contextualizing established theories of technology acceptance within the unique mandatory use context of anti-doping. While frameworks like the unified theory of technology acceptance and use [UTAUT; (63)] or risk-benefit models (41) are well-established, their dynamics shift significantly when choice is eliminated. Our findings demonstrate that in such an environment, perceptions of procedural justice, fairness, and trust become more relevant determinants of perceived legitimacy, rather than perceived usefulness alone (33). This generates practical insights for regulators, ADOs, and technology providers on how to design, communicate, and implement RSS in ways that build trust (66), address uncertainties (67), and foster acceptance among those most directly affected (33). The empirical study shows how mandatory implementation transforms user concerns from questions of voluntary adoption (“Should I use this?”) to questions of risk and fairness (“How can I safely comply with this?”).

Methodologically, the study presents a comparative qualitative design for examining technology adoption in a multi-stakeholder environment. By systematically interviewing and contrasting the perspectives of two distinct user groups (athletes and DCOs) with varying levels of experience, the interview approach provides a template for uncovering both shared concerns and divergent, role-specific tensions. Second, we demonstrate the value of a process-oriented template analysis. Structuring our analysis along the three distinct phases of the RSS procedure (preparation, collection, finalization) allowed for a granular investigation that moves beyond general perceptions to reveal phase-specific challenges and opportunities.

### 5.2 Practical implications for anti-doping practice

This study offers several practical implications for ADOs, WADA, and technology developers involved in the design and implementation of RSS.

First, our findings underscore the need for a user-centered design approach. The concerns raised by both athletes and DCOs are not definite barriers, but actionable design requirements. ADOs should move beyond top-down implementation and actively involve these user groups in the development and iterative refinement of remote processes. For example, athletes' feedback on logistical burdens suggests the need for multi-kit solutions and flexible delivery options. Similarly, DCOs' concerns about observability can guide the utilization of better virtual supervision tools and protocols. Their shared preference for DBS sampling in the remote setting provides a clear mandate to prioritize and invest in the scaling of this less invasive and more user-accepted sampling method.

Second, the study highlights the need to define a digitally enabled chain of custody. The “logistical black box” of sample shipment is a critical point of failure for both user groups' trust. ADOs and WADA must develop a robust, legally defensible framework for this hybrid physical-digital process. This includes, for example, establishing clear protocols for the unobserved “last mile” of shipping, mandating integrated digital tracking and hand-off confirmations, and defining clear liability rules in case of logistical failures. Creating such a framework is essential to address athletes' fears of unfair sanctioning and DCOs' concerns about process integrity.

Third, a successful transition to RSS requires investment in training and role redefinition. For DCOs, it requires developing new competencies in remote supervision, virtual communication, and digital evidence assessment to support their evolving professional identity (40). For athletes, clear, accessible, and multilingual instructions and support channels are needed to reduce their anxiety and the risk of procedural errors. By proactively managing this change, ADOs can foster a sense of competence and affirmation, rather than resistance.

Finally, ADOs must recognize that RSS implementation is not merely a technological challenge, but a challenge of trust management. The perception of fairness is paramount. By transparently addressing the identified risks, providing robust support, and demonstrating that the system is not only efficient but also just, ADOs can build the legitimacy needed for RSS to become a sustainable and accepted tool in anti-doping testing regimes.

### 5.3 Limitations and future research

This qualitative study offers process-level insights, but several limitations temper transferability. The sample is purposive and limited to elite athletes and DCOs from a subset of countries and is not statistically representative across sports, genders, impairment categories, or regulatory environments. Views of distinct stakeholder groups like Para-athletes, athletes from low-tier signatory countries, or minors facing distinct constraints are only partially captured and likely underrepresented. It should be noted that in the sample, only a minority of athletes and DCOs interviewed had actual experience with remote sampling procedures in pilot projects. Therefore, the derived themes are based primarily on hypothetical scenarios that do not capture lived realities and experiences associated with the application of remote sampling. This likely introduces selection effects and contextual bias. Also, recall bias is possible when athletes and DCOs refer to past pilot experiences, or hypothetical scenarios.

The analysis relies on template analysis (44, 59) that utilizes the three RSS process stages as structuring and organizing elements to develop themes from the interview material. While this approach allows us to distinctly analyze and understand considerations and views of both user groups in the respective process stages, this style of analysis risks fracturing broader considerations concerning multiple process stages. Interviews captured perceptions such as missed tests or delivery failures, which were not linked to objective outcomes. Therefore, such considerations concerning procedural integrity remain perceptual. In addition, the technological landscape is evolving. Findings may be tied to specific devices, app versions, or vendor workflows, limiting generalizability to other RSS designs that may emerge in different technology paradigms.

These limitations directly inform several avenues for future research. First, future studies should purposefully target underrepresented and vulnerable user groups. Dedicated research is needed to understand the specific challenges faced by e.g., para-athletes, who may require assisted sampling, and by athletes in low-tier signatory countries, who may face greater financial, technological and logistical hurdles. Examining the perspectives of minor athletes and their guardians is also critical to ensure that RSS protocols are ethically and practically sound for all segments of the athlete community. Following up on this, the scope of inquiry should be expanded to a broader ecosystem perspective. While our study focused on the core users of the RSS, future research should investigate the downstream impacts of RSS on other key stakeholders. This includes laboratory personnel, who must adapt to new sample types [e.g., DBS samples collected in new self-sampling devices (22);] or quantities, and potential quality variations; anti-doping administrators, who manage the logistical back end; and the legal and results management professionals who must defend the integrity of remote samples in a judicial context.

A further avenue concerns sample privacy along the unobserved shipment phase. Even when packages carry only coded labels, the biological material itself remains theoretically re-identifiable through downstream genetic or biomarker analysis if intercepted. While this risk also exists in traditional in-person testing, the decentralized and athlete-handled shipment under RSS may amplify exposure. Future work should formally assess threat models for RSS shipment and evaluate technical safeguards, such as tamper-evident packaging with cryptographically signed tracking IDs and certified medical-grade logistics partners with auditable chain-of-custody records. Such analysis would provide a more holistic assessment of the viability and long-term implications of remote anti-doping.

## 6 Conclusion

As a response to the logistical challenges of traditional in-person doping control, remote sampling offers a promising technological solution, but its implementation is far from straightforward. This study reveals that successful implementation is not a simple matter of technological feasibility. By analyzing the perspectives of athletes and DCOs, we uncover divergence in perceptions of the remote sampling procedure, which we conceptualize as a shift from DCO control to athlete burden. While athletes perceive the transfer of responsibilities as an unjust personal burden of risk and logistical effort, DCOs view it as a critical loss of professional control over procedural integrity.

This core tension shapes how each group evaluates the system's legitimacy within the mandatory anti-doping context. Our findings demonstrate that user acceptance hinges on resolving this conflict. The path forward is not to choose one perspective over the other, but to design a system that consciously reconciles both. A successful RSS must mitigate the athletes' burden through user-centered design, robust support, and clear procedural safeguards, while simultaneously empowering DCOs with reliable tools for verification and control. Ultimately, the future of remote anti-doping depends on the ability of ADOs to implement a system that is not only technologically sound but also perceived as legitimate and trusted by those at the very heart of its operation.

## Data Availability

The raw data supporting the conclusions of this article will be made available by the authors, upon reasonable request.
